# The unique contribution of e-cigarettes for tobacco harm reduction in supporting smoking relapse prevention

**DOI:** 10.1186/s12954-018-0237-7

**Published:** 2018-06-20

**Authors:** Caitlin Notley, Emma Ward, Lynne Dawkins, Richard Holland

**Affiliations:** 1 Norwich Medical School, Norwich Research Park, University of East Anglia, Norwich, NR4 7TJ UK; 2 Centre for Addictive Behaviours Research, School of Applied Sciences, London South Bank University, 103 Borough Road, London, SE1 0AA UK; 3 Leicester Medical School, Leicester, UK

**Keywords:** Electronic cigarette, Vaping, Smoking relapse prevention, Qualitative

## Abstract

**Background:**

We have little understanding of how vapers use e-cigarettes beyond cessation. E-cigarettes may have a role to play in reducing the health-related harms of tobacco smoking, through not only assisting smoking cessation attempts but also supporting long-term abstinence from smoking. However, there are fears that vaping may lead to the ‘renormalisation’ of smoking type behaviours. This study aimed to explore patterns of use and reported experiences of vapers quitting smoking using an e-cigarette in relation to long-term smoking status (abstinence or relapse).

**Methods:**

A purposive sample of 40 UK vapers was matched to a sampling frame of demographic characteristics from a representative sample of UK quitters. Following full informed consent, semi-structured qualitative interviews were conducted. Data were thematically analysed by two members of the research team. Final thematic analysis was verified and agreed by consensus.

**Results:**

The sample self-reported long histories of tobacco use and multiple previous quit attempts which had eventually resulted in relapse back to smoking, although a small but important group had never before attempted to quit. Initiating e-cigarette use was experienced as a revelation for some, who were quickly able to fully switch to using e-cigarettes as an alternative to tobacco smoking. For others, periods of dual use or smoking relapse combined with attempts at vaping that were not initially satisfactory. Many of these chose a cheaper ‘cig-a-like’ device which they found to be inadequate. Experimentation with different devices and different setups, over time, resulted in some ‘sliding’ rather than switching to vaping. This involved periods of ‘dual use’. Some settled on patterns of vaping as a direct substitute of previous tobacco smoking, whereas others reported ‘grazing’ patterns of vaping throughout the day that were perceived to support tobacco smoking abstinence.

**Conclusions:**

Our data demonstrates that e-cigarettes may be a unique harm reduction innovation for smoking relapse prevention. E-cigarettes meet the needs of some ex-smokers by substituting physical, psychological, social, cultural and identity-related aspects of tobacco addiction. Some vapers reported that they found vaping pleasurable and enjoyable—being more than a substitute but actually preferred, over time, to tobacco smoking. This clearly suggests that vaping is a viable long-term substitute for smoking, with substantial implications for tobacco harm reduction.

## Background

E-cigarettes may have a role to play in reducing the health-related harms of tobacco smoking through not only assisting smoking cessation attempts but, perhaps more importantly, supporting long-term abstinence from tobacco smoking. A review of the latest available evidence suggests that e-cigarettes are at least 95% less harmful to health than tobacco smoking [1]. E-cigarettes have become the most popular aid to quitting smoking in the UK [1]. This is perhaps particularly so for the general population not wishing to seek formal support for smoking cessation from a health professional. Indeed, it is for this population of non-help seekers that the harm reduction role of e-cigarettes may be most clearly realised. There is now substantial support for vaping as a harm reduction approach and an alternative to tobacco smoking from UK Medical [2] and public health bodies [1, 3].

However, the use of e-cigarettes for smoking cessation, and particularly longer-term use, remains controversial. The UK takes a relatively permissive stance, but vaping is still banned in many countries [4]. The long-term health effects of vaping are unknown and may not be clearly recognised for many years, since most e-cigarette users are ex-smokers, and thus, disaggregating the health impact of vaping from previous smoking is difficult. Although studies are beginning to show that the health impact of vaping may be comparable to NRT use [4], there are concerns from some public health perspectives that vaping may ‘renormalise’ ‘smoking-like’ behaviours. This may actually encourage more people to smoke or those who have quit to potentially relapse [5]. Despite vaping being recognised as a less harmful method of using nicotine than tobacco smoking, addiction to nicotine is maintained, and this can be a cause for concern [6]. E-cigarettes are a consumer product, which possibly has created some unrest amongst the medical community, who may perceive medicalised forms of nicotine (NRT) as superior or safer harm reduction approaches to the continued use of nicotine [7]. The concept of addiction itself is morally laden, with many health professionals, and indeed consumers, having a view that the avoidance of any addiction at all is preferable [7]. Thus, the longer-term use of e-cigarettes in relation to tobacco smoking status is a controversial area, due to the inherent duality of e-cigarettes as both offering potential for long-term smoking abstinence yet supporting a continued addiction to nicotine [8].

Prior to the widespread use of e-cigarettes, longitudinal studies exploring smoking cessation attempts in relation to long-term outcomes suggested that although many smokers managed to initially quit, over time, relapse to tobacco smoking was common [9]. Research suggests a range of psychosocial factors at play in contributing to relapse, including physical addiction (craving), behavioural cues to smoke (e.g. for the ‘habit’), social and environmental cues to smoke (being with other smokers, associating smoking as social relaxation) [10–17] and concepts that cut across psychological and social domains, such as relapse as signifying a regaining of the lost identity that was available as a smoker [18, 19]. Initial lapse to tobacco smoking has in the past been shown to be highly predictive of full relapse [20]. However, emergent qualitative evidence suggests that this association may potentially be less strong in the context of e-cigarette use [21]. We have little understanding of how vapers use e-cigarettes beyond cessation to specifically avoid long-term smoking relapse. There is also concern that vapers may continue to ‘dual use’ e-cigarettes alongside continued tobacco smoking. This study sought to purposefully recruit for in-depth interviews people in the general population (not accessing specialist smoking cessation services) who attempted to quit smoking using an e-cigarette, specifically exploring how reported patterns and trajectories of use over time may or may not support long-term smoking abstinence.

## Methods

We sought to answer the research questions: ‘what is the experience of e-cigarette use over time?’ and ‘what are vapers’ reported experiences of either tobacco smoking abstinence or relapse?’ A qualitative approach was particularly suitable in this context, where existing theory is outdated in the context of a fast-moving consumer market establishing e-cigarettes as integral to the majority of smoking quit attempts.

For recruitment, we defined smoking relapse as ‘a successful smoking quit attempt of at least 48 h, followed by a relapse (more than five instances of reported lapse) to tobacco smoking’. This was an inclusive definition in order to capture both early and late relapsers, whilst excluding dual users and triallers (those who use e-cigarettes alongside tobacco smoking without making a serious quit attempt). This gives us a practical and clear definition by which to differentiate those e-cigarette users who we can define as ‘relapsed to smoking’ as compared to those ‘abstinent from smoking’.

The study comprised qualitative interviews using purposive sampling. Participants were initially recruited using established personal networks of the research team, via self-referral through advertising in local, national and social media, and snowballing to seek referrals from interviewees. From eligible referrals, a purposive sample of 40 UK vapers was matched by gender and age to a sampling frame of demographic characteristics from a representative sample of UK quitters (Table 1). This sample size was adequate to reach saturation of experiences and key themes [22]. Cross-sectional semi-structured qualitative interviews were conducted between September 2016 and May 2017.

**Table 1:** Demographics of the interview sample (*n* = 40)

	Participants	
	%	*n*
Gender (female)	50.0	20
Ethnicity		
White British	92.5	37
White European	7.5	3
Occupation [30]		
Managers, directors and senior officials	7.5	3
Professional occupations	20	8
Associate professional and technical occupations	12.5	5
Skilled trades occupations	5	2
Caring, leisure and others services occupations	5	2
Sales and customer service occupations	7.5	3
Process, plant and machine operatives	2.5	1
Stay at home parent	2.5	1
Full-time student	15	6
Retired	7.5	3
Age		
Age range	21–70	
Mean age (SD)	41.2 (14.0)	

Participants gave written consent for interviews (face-to-face or telephone). Semi-structured guides took a narrative approach to explore participant histories of tobacco smoking and prior quit attempts, through to e-cigarette initiation and whether this was part of an intended quit attempt. Patterns of e-cigarette use were explored. We asked participants to describe the devices they used and the nicotine e-liquid (flavour and strength) they had started with and then to describe in detail any changes in patterns of use or vaping setups over time. Simultaneously, we asked participants to describe the situations and experience of any lapse or relapse to tobacco smoking where this had occurred. We asked participants to reflect on their future intentions and identity-related aspects of vaping. Interviews were transcribed verbatim and anonymised. Participant codes used to reference quotes refer to participant’s gender and age (e.g. ‘F24’ for ‘female aged 24’). Transcripts were thematically analysed [23] systematically case-by-case independently by both CN and EW, with a 10% independent coding check undertaken, whereby both analysts coded the same transcripts and compared coding. The analysis was discussed at regular team meetings, and anomalies were agreed by consensus. Individual case summaries were written to facilitate cross-case comparison, and pathways diagrams were plotted to illustrate participant journeys through smoking cessation.

## Results

Participants’ mean age was 41 (SD 14.0, range 21–70) (Table 2), and gender was split equally. All participants identified as White British or European, 16 were employed in managerial, professional or technical occupations. 33 were recruited in East Anglia with the remainder located across other parts of England. Vaping experience varied from starting two weeks before the interview to seven years. 31 participants were vaping and abstinent from tobacco (19 had reported lapses), six participants had relapsed (five dual using both tobacco and vaping) and three were no longer using either e-cigarettes or tobacco.

**Table 2:** Gender and age profile of achieved sample compared to proportion of past-year smokers who had made at least one attempt to quit smoking, surveyed between 2015 and October 2016, in the Smoking Toolkit Study [1]*

	Achieved sample		Target sampling frame		
	%	*n*	STS %	STS *n*	Target *n*
Gender					
Male	50.0	20	51.4	1205	21
Female	50.0	20	48.6	1140	19
Age (years)					
16–24	12.5	5	19.1	448	8
25–34	20.0	8	26.0	609	10
35–44	30.0	12	19.4	455	8
45–54	17.5	7	17.6	412	7
55–64	15.0	6	10.8	253	4
65+	5.0	2	7.2	168	3

*STS* Smoking Toolkit Study data

### Pathways through smoking cessation and initiating vaping

**Fig. 1:**
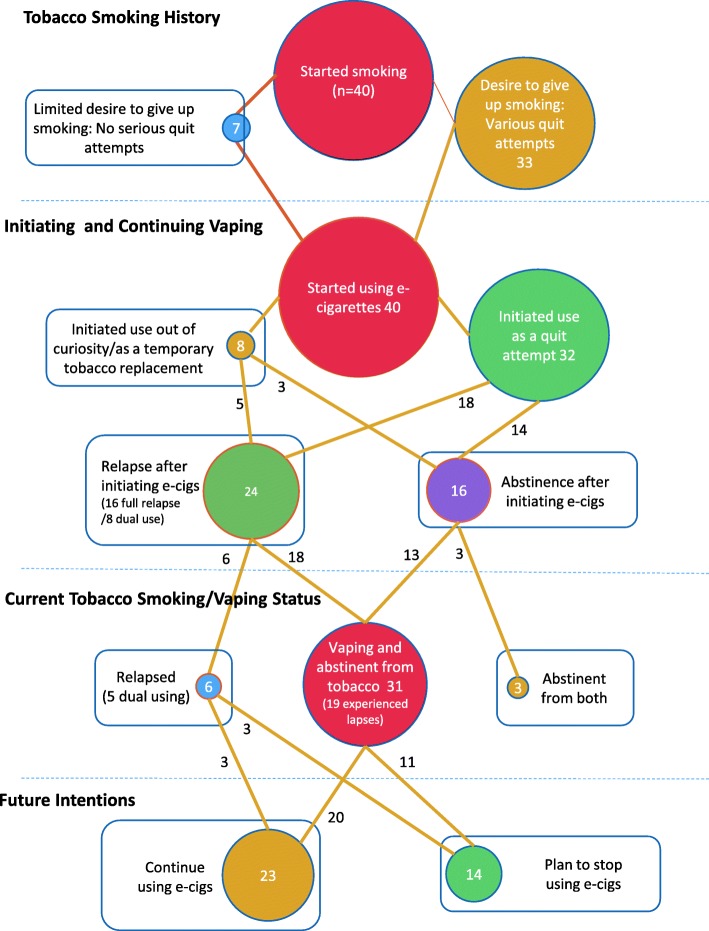
Pathways through smoking cessation and e-cigarette use

Figure 1 provides a summary of the total sample encapsulating reported pathways through initiating e-cigarette use and quitting tobacco smoking.

Most of our sample reported long histories of tobacco smoking and multiple previous quit attempts However, a minority (seven people) had never seriously attempted to quit smoking. These people stated that they enjoyed smoking and had no particular desire to quit:

I’d never wanted to enough, you know, obviously I’m very aware of the health effects and what not, but I always just enjoyed it too much, and that always took precedent over the health side of things, yes, so I’d never tried, cos I didn’t want to enough, and I knew that you really have to want to do it (F24)

#### Accidental quitters

Those in our sample who stated that they had not intended to quit smoking had tried vaping on a whim or because they been offered by friends. In these cases, individuals found that they liked it or saw that it offered potential as a substitute for smoking:

one evening I went to the local shop, which is very close to here, to get some cigarettes, and they had run out of the brand that I smoked, and I don’t know why, but instead of choosing another brand which I could of done, and occasionally had done in the past, I said ‘oh well, I’ll try one of those e things, I’ll just try one’ and so I bought it, it was a disposable thing and looking back on it, it didn’t taste very nice, it had a sort of metallic taste to it, and I know that it wasn’t a brand that I would now seek out, but I did, that’s what I did, I just bought it and brought it home, and I said to my wife that evening, ‘you know, this is all right, it’s sufficiently satisfying’ (ok) that I think I might investigate this (ok) and I have never smoked tobacco since that day (really), not one drag, and I have never felt that I wanted to (M63)

The narrative here demonstrates vaping is experienced as a ‘revelation’ in terms of quickly and easily substituting previous smoking behaviour. In this narrative, the smoking quit attempt is ‘accidental’:

it felt like I was smoking, so I didn’t have to kind of think up displacement activities, I didn’t have to find something else to do with myself, I could do exactly what I’d always done, just with a slightly different device, and yes, I really, really took to it, and within five days I’d chucked out the last of my cigarettes…but you know within 5 days I’d stopped completely without meaning to (F38)

Many people discussed how they felt that e-cigarette use was a ‘no pressure’ approach to quitting:

it was a natural progression because I enjoyed it, it was easy to do, I didn’t even think about, like I said, if I put myself under pressure I probably fail at it, and think ‘oh, I have to do this’, but as I didn’t, I just, it just organically happened really for me (F34)

The success of vaping as an aid to quitting smoking can therefore be understood both in terms of the satisfying physical substitution of nicotine delivery, but also the psychological and social relief of not declaring oneself as ‘quitting smoking’, thus not setting oneself up to potentially fail. This is in contrast to the ‘success/failure’ narrative experienced in the past when attempting to quit smoking by other means:

absolutely lousy, feel like a complete failure (right)… you know you’ve put in an awful lot of effort, you’ve gone through all of the struggle of getting off of an addiction, and to fall so easily into an addiction that is very, very easy to fall back into (yes, yes)…you know it’s an awful feeling (M44)

#### Purposeful quitters

Most of our sample had initiated e-cigarette use because they actively wanted to quit smoking:

gum wasn’t going to cut it basically and the patches just didn’t seem like a good idea to me I didn’t think I wasn’t to make a real attempt and patches and gum weren’t going to work so actually try something that may possibly work (M30).

For these people, e-cigarette use was experienced as substantially different from other smoking cessation support. There were important strategies employed that may have impacted on the outcome. Some people tried to equate a number of cigarettes smoked to liquid strength, usually with the guidance of a shop [24] or peers who were experienced vapers:

The guy in the shop spoke to me about, like, how many I smoked a day, how often I smoked, what I was smoking, that kind of stuff and cos you can go up to 24 mg, but he said no because I was only on ten [cigarettes a day] (right) so 18 was probably the best one to start on (M36)

#### Switching

Many of our sample switched quickly and completely from smoking to vaping:

having tried so often and failed so often and not having got on with anything else, I was expecting it to be really really hard (ok) but I think the combination of finding something that reproduced the experience but without so many of the carcinogens was the thing that worked for me. So I found it remarkably easy, it sounds silly to say, but having tried and failed so often, having been a smoker for such a long time I didn’t find it hard. (M53)

Participants described how easy it was to quit using this method and how they enjoyed vaping in its own right. This suggests that vaping offers something additional to nicotine replacement—it is a pleasurable alternative.

I think the ways of giving up that as far as I know are on offer at the moment with, you know, gum and patches and stuff like that, that helps with the physical cravings, but it doesn’t help with the habits, and it doesn’t help with the feeling of breathing something in and breathing smoke out, which, you know, which is such a fundamental part of smoking that, you know, I think I wouldn’t have given up if those had been the only alternatives offered (F38)

For others, vaping was viewed as a continuum with smoking, as opposed to a method of smoking cessation. For these people, using an e-cigarette was not seen as giving up smoking, just smoking in a different way. There was a level of comfortableness with addiction to nicotine, seeing it as acceptable as a harm-reducing way of using nicotine:

To actually have [nicotine] in the form of an e-cigarette can be more effective than nicotine chewing gum or nicotine patches cos they’re basically a method of stopping smoking and stopping the addiction to nicotine aren’t they? Whereas e-cigarettes, you’ve still got the addiction to nicotine but the method that you’re ingesting it is can be as, you know, not far off as satisfying as having a cigarette (M49)

#### Sliding

Employing the ‘no pressure’ strategy, some people found themselves naturally ‘sliding’ towards smoking cessation—initially ‘dual using’ both tobacco and e-cigarettes, but over time coming to use e-cigarettes more and eventually completely vaping and being tobacco-free:

I didn’t stop like one day… as one went down and the other went up (ok) so it sort of replaced one with the other rather than cold turkey…I think once I started to do this because it was around, and actually like, if you’re at home I could smoke it (the e cigarette) whereas the other one (tobacco) I’d have, I didn’t really smoke at home, I would have had to go outside or whatever (yes) it was easier to smoke it (the e cigarette), so that made it better (yes) and then at some point I thought ‘I’m not buying another packet’ (of tobacco cigarettes), so then you sort of force the issue and you’re not smoking much at all (F36b)

Sliding appears to be a novel experience in terms of smoking cessation, suggesting an important route into fully quitting tobacco smoking for some people, particularly those who dual use or do not actively want to quit smoking.

#### Relapse after initiating

Despite initial success, that for some people was easily maintained, a majority of people either fully relapsed [16] or dual used [8] after purchasing their first device. For some, this was because they were not actually initially attempting to give up smoking. For others, brief lapse to tobacco smoking occurred due to social or emotional reasons. However, importantly, lapse for these people did not appear to be as catastrophic as it may have been in the past, as it did not necessarily lead to full relapse [21].

However, for many, relapse/dual use occurred after initiation due to inferiorities in the first device purchased. Although a few people in the sample did manage to maintain full smoking abstinence using a device that they did not find fully satisfying, most people did not manage to maintain abstinence initially. The reasons people gave for their first device being inferior were mostly related to first generation or ‘cig-a-like’ type devices, which were generally experienced as unsatisfying:

I think they had them in the shop down the road, just like the ones that looked like a cigarette, so I tried that to start with, but they’re not very good, I don’t think, and if you’re seriously thinking of you’re, like swapping that for cigarettes, for those sort of ones I think you try them, and think, ‘actually they’re nothing like it’ and (yes) and I think that puts a lot of people off (F46)

Others reported that the first devices they tried were inadequate or continually malfunctioned:

I did try e-cigarettes before in the past, about early 2010 let’s say, but I didn’t really like them. They just didn’t work too well, they were spitting liquid nicotine in your mouth and awful stuff like that, so that was definitely a no go for me. (M22)

### Avoiding relapse to tobacco smoking

#### Finding an effective and satisfying vaping setup

Through trialling different setups, most people in our sample had eventually achieved abstinence by getting the right vaping setup and learning to vape in patterns that met their needs. For some, this was a personal quest and a process of trial and error, whereas others sought support from other vapers, forums or vape shops.

just picking up an e-cigarette from a newsagent and taking a puff and thinking no, that’s no good, isn’t the end of the story, because there are different brands, different tastes, different strengths and flavours, you can get it right, and it can be a substitute (M63)

Participants had strategies that they had learnt over time to ensure their e-cigarette setup was reliable:

The batteries, I weren’t too sure how long they would last and when I first started I thought “oh my god what about if that run out?” and so I bought another one exactly the same (ok) so I have one battery on and one already charged up. (F60)

#### Developing patterns of use that are helpful in maintaining abstinence from smoking

Participants described varying individualised patterns of use of their e-cigarette. The key point is that, over time, individuals came to vape in patterns that they found acceptable, enjoyable and that were sufficiently satisfying so that they did not experience strong cravings for tobacco. For some, patterns of vaping directly replicated previous patterns of smoking:

it was beside my car from when I got in from work, sit down, cup of tea and the e-cigarette, so exactly when I would have previously have had a couple of cigarettes, and then again during the evening after a meal, coffee and the e-cigarette, so exactly when I would have had two or three more, watching television during the evening, and so on (F62)

However, for many, patterns of vaping were very different from previous patterns of smoking. Particularly, people vaped in places they would not previously have smoked (in the house, in bed, in the car, ‘in secret’ at work). The majority ‘grazed’ on their device throughout the day and could not really say how often they used their e-cigarette or how many puffs they took. This was contrasted to smoking:

I feel like I’m grazing on it constantly (yes), whereas with a cigarette it’s, you know, when it’s done you’ve had enough because it’s finished, whereas with [e-cig] I never really know when I’ve had enough I suppose. (F36)

For some, the ‘grazing’ pattern of vaping meant that they tended to vape little and often, which may well have impacted on nicotine levels in the body, such that people used the e-cigarette to avoid craving through nicotine withdrawal:

I use it all day long, whenever, I suppose nicotine withdrawal is kicking in, but I’m not doing it for the numerous minutes that smoking a cigarette can take, so it’s often two or three inhales, back in the pocket of the handbag, forget it for a little while (F62)

Participants’ beliefs towards grazing behaviour were generally positive:

That was the annoying thing about cigarettes, they stop and you’ve got to then say right I’m not going to light another one I’m going to leave it for a certain amount of time, so you immediately start withdrawing as soon as you’ve stubbed one out. You’re kind of like ‘I want another one now, but I’m not going to’ and then ‘oh I’ll give in, I’ll have another one’, but with this I have one little puff every now and then. (F52)

However, some were perturbed by the grazing pattern of use and worried that this meant they may be increasing their addiction to nicotine:

It’s so easy and less invasive I probably, yes I don’t know, if I vape more than I would smoke (M40)

### Potential factors in supporting smoking abstinence

#### Replication of smoking

For those at the time of interview reporting vaping and abstinence from smoking, vaping was discussed as similar to smoking in meeting nicotine addiction needs, thus assisting the individual to avoid tobacco smoking relapse:

the vaporiser is a very similar form of receiving the nicotine whereas if you just stick a mint (NRT) in your mouth that’s completely different to smoking (M37)

Ex-smokers also used their e-cigarette to meet psychological needs, e.g. e-cigarettes were used as part of the pattern and routine of everyday life, as a direct substitution for when cigarettes might previously have been used:

even the addiction, like the nicotine necessarily, I don’t think it’s the habit of it, and the like, it’s very embedded in my psyche, like, I enjoy going and standing outside in the evening and having a cigarette, it’s not even, I mean the cigarette is a big part of it, but it’s the habit and the like, the ritual of it as well (F24)

E-cigarettes were enjoyed due to the habitual aspects of vaping that mirrored previous smoking behaviour. The ‘hand-to-mouth’ action, holding something in the hand, breathing vapour into the lungs and blowing our vapour were all mentioned as important behavioural aspects that potentially contributed to the prevention of tobacco smoking relapse:

part of my problem has always been it’s a habit and it’s having it in your hand and sticking it in your mouth so it’s a replacement it’s a direct replacement for doing, putting something in your hand, and it generates smoke and I know that sounds daft but the e-lite thing didn’t make that much smoke, and it’s weird when you’re smoking a cigarette for it not to make smoke, so it sort of ticked all those boxes and it actually tasted nicer than a cigarette (F36)

Socially, vaping was experienced by many as an alternative to smoking. Unlike with previous smoking quit attempts, vapers felt that they did not have to distance themselves from their existing social networks in order to avoid smoking relapse:

some people get into that routine I suppose, where they still quite like to have that time away from their desk and walk outside, cos that’s what they were used to when they were smokers, so they’ll go and stand outside in the smoking section, but they’ll vape (M37)

Vaping facilitated a sense of belonging to a specific social group for some:

[There’s] a big community […] I see fellow vapers in town we just we just sort of give each other a look, we look at each others mods, it’s sort of like the classic “oh you’re a vaper, hi” the whole community is all one. [F21]

#### Identity

The social acceptance of vaping suggests that vaping met the identity needs of some of our sample. For those who had previously strongly associated with a smoker identity, vaping offered an alternative attractive identity:

I can well see that, well I know people that are quite content to be vapers for the rest of their life, you know, they haven’t even got any interest in coming off the nicotine and they’re at a level that they’re comfortable with, and they do view themselves as vapers, and they’re going to remain vapers, because that’s what they’re happy with, you know, so from that element, you know, there’s an awful lot of diversity out there, there’s a lot to keep them interested, and I don’t view that as a negative thing, you know, it’s a good thing (M44)

Here, it is clear that there are numerous potential vaping identities available within the category of ‘vaper’. For some, associating with one of these vaping identities clearly supported continued vaping and may have played a role in tobacco relapse prevention. However, for others, the ‘vaper’ identity was minimised in favour of an ‘ex-smoker’ identity, positioning oneself differently from those who identified with the social and cultural aspects of vaping:

[I see myself as] an ex smoker. I would say an ex smoker I think, but there’s still the nicotine addiction there. I don’t know if I would really call myself a vaper. I vape but, you know, it’s still something that I, yes I don’t sometimes. I’ll always be a smoker, it’s something that’s never going to sort of go away, but now I can start saying I’m actually or become an ex-smoker (F46)

#### Pleasure

As well as substituting physical, psychological, social and cultural elements of previous tobacco smoking behaviour, vaping was frequently discussed as enjoyable and pleasurable in its own right—sometime above and beyond how tobacco smoking was perceived:

it often smells quite pleasant and the vapour dissipates quite quickly, it doesn’t stick to clothing, so you know I think people have got a better perception of it rather than tobacco smoke (M37)

Flavours and smells, the sensory aspect of vaping, were an important and pleasurable aspect:

it’s probably one of the most important things in a way, in a sense, that if it was flavourless I don’t think it would really have, it’s actively pleasurable, it’s a nice thing. It’s that bit that means it’s fundamentally different in my mind between a patch or chewing gum or the spray. (M39)

#### Practicalities

Practical aspects, such as reduced cost compared to smoking, accessibility and ease of use, were important in discussing continued e-cigarette use:

if you’re spending a lot of money on cigarettes it, you know, it’s a massive saving when you switch to vaping (F40)

Accessibility was multi-faceted. E-cigarettes were enjoyed because of their accessibility for everyday use and convenience:

It’s a small little tight unit that fits in my pocket. Yes just it just does everything and its tiny, I think that’s the thing that works for me, it’s small, compact and it’s not much hassle. (M26)

Partly, the convenience aspect was enjoyed as vaping could be easily and discreetly incorporated into everyday situations:

I didn’t have to go outside and stand out there in the cold for five or ten minutes while I smoked the cigarette. I could just sort of, when the wife’s not looking (yes) have a quick puff (M70)

#### Perceived health benefits

Participants discussed multiple perceived health benefits that corresponded to the health benefits of quitting smoking, as they remained abstinent from smoking by vaping:

I could tell that my sort of breathing was a lot better, my skin was good, my teeth were good, hair and nails were growing well, and generally just felt a lot better in myself (F27)

As well as improved respiratory function, which was frequently discussed, marked improvements in sensations of taste and small were noted:

In the first sort of six weeks I noticed the usual sort of, blimey what’s that nasty smell, I never used to smell that before, but yes the usual taste and smell thing. (M58)

### Future intentions

Some ‘invested vapers’ saw themselves as invested in vaping as a hobby or were interested in the technical aspects of it. They viewed it as a fairly significant part of their life. It had become more than a smoking cessation aid and was an enjoyable pastime in its own right. Therefore, they had no intention to quit:

I have no wish to wean myself off… I don’t look at it now as a keeping me off the cigarettes, cos I don’t want a cigarette at all, so it’s not really keeping me off the cigarettes, it’s a hobby now, and a social thing, and thats, I will carry on vaping because, you know, it’s a hobby and a social thing (M67)

Others were really enthusiastic about how vaping had enabled them to easily stop smoking. These ‘enthusiastic switchers’ felt vaping had been successful mainly for its ability to replicate smoking in terms of satisfying cravings and the enjoyment they got from smoking:

I’m addicted to nicotine, so yes, yes, I’m a vaper but I’m not one of these big beardy weirdy hipsters who just spends all day long, you know, going harping on about this new thing and that new thing, and this massive cloud, and so I’m not like that, I’m an ex-smoker who is addicted to nicotine, so I won’t be giving it up because it’s got nicotine (M41)

This group was happy to continue to vape. They were concerned that if they reduced their vaping, they would be vulnerable to tobacco smoking relapse. Therefore, although they were not fully invested in a vaping identity as with the above group, they discussed no intention to quit vaping.

Many invested vapers and enthusiastic switchers were completely comfortable having a dependence on nicotine:

I’m kind of a little resigned to having a nicotine addiction. That seemed a particularly good way of satisfying it without going back to sort of smoking excessively. (M49)

However, a very different group, ‘nicotine quitters’ were people who had quit e-cigarettes or were intending to. They mainly viewed e-cigarettes as a smoking cessation aid to help them eventually quit nicotine altogether:

I think some people see it as more like a hobby, it’s like, now I’m not smoking I’m going to vape, but I see it more like cutting down, using it to quit, and then I want to get rid it, and then only use it if I need it for social occasions (F22)

In contrast to the other groups, many of those who wanted to quit e-cigarettes wanted to because they were uncomfortable having a dependency:

I don’t like the idea of being addicted to anything. It’s not, I don’t think nicotine is 100% safe, I know it’s a lot better vaping than you know smoking cigarettes, so you know I’m quite pleased we’ve done that bit, but I just feel so silly having this prop. (F59)

## Discussion

Our findings demonstrate that e-cigarettes may be a unique harm reduction intervention for smoking relapse prevention. Particularly, vaping was shown in our data to be an attractive option offering a route into smoking cessation for those people who did not initially want to stop smoking, or have found it difficult to stop smoking using other means of cessation support. Many of these people would not consider seeking formal support from a healthcare professional to quit smoking, but might try vaping on a whim or because they are offered, and subsequently go on to ‘accidentally quit’. This point is critical not just for initial smoking cessation but for supporting long-term abstinence from tobacco smoking. Vaping was perceived qualitatively differently to other forms of smoking cessation support, offering a pleasurable and enjoyable alternative to smoking. Vaping was discussed as meeting the needs of our sample of ex-smokers by substituting physical, psychological, social, cultural and identity-related aspects of tobacco addiction. Indeed, for many, the potential of vaping as ‘better than smoking’ suggests that sliding or switching to vaping is a viable long-term substitute for smoking.

In line with the principles of a harm reduction approach to ‘treatment’ of addictive behaviours, our findings suggest that many smokers enjoy and do not wish to quit smoking unless there is an equally enjoyable alternative. In this respect, vaping is attractive. In first trying vaping, those who initiated use experimentally or on a whim often reported periods of dual use, corresponding to other qualitative evidence [25]. Our data on patterns of vaping over time suggest that ‘sliding’ towards cessation without purposefully meaning to quit smoking was an important pattern. Thus, positioning dual use as problematic may be premature; instead, we might helpfully see dual use on a trajectory to smoking cessation and support accordingly [26].

A discourse of the importance of pleasure in tobacco harm reduction is emerging [27]. This was a strong theme in our data. Pleasure as an emotion, desire or simply a state of being intersects with the concept of identity, since humans naturally engage in pleasurable or rewarding behaviours that bolster self-esteem and confirm a positive sense of social identity [28]. It has been demonstrated that identity change is a prerequisite to successful long-term smoking abstinence [29–31], and where the ex-smoker identity is disrupted, consequentially, relapse may occur [18]. Importantly, our findings demonstrate that vaping offers the availability of a range of alternative identities [32] that are culturally and socially acceptable to ex-smokers.

Our data detailing varied but patterned pathways through initiating vaping clearly suggest that smoking cessation by vaping is a process that unfolds over time. Individuals described how they frequently experimented with first generation ‘cig-a-like’ devices, which were generally found to be ineffective, before finding a vaping setup that suited their individualised needs and preferences. For practically supporting ex-smokers to quit smoking and to prevent relapse, this suggests that vapers need ongoing support and advice, firstly to find a suitable vaping setup and, secondly, to learn to use the device in patterns that are acceptable and that substitute the habit of smoking sufficiently, from a physical, psychological and social perspective. Finally, vapers need ongoing support to troubleshoot problems, such as difficulties with device function, in order to prevent potential tobacco smoking relapse, as continuing to vape is associated with continued abstinence from smoking [33].

Theoretically, our findings shift the paradigm of thinking related to smoking relapse prevention, through illuminating the unique ability of e-cigarettes to substitute psychosocial, psychological and social aspects that were previously enjoyed about smoking. Development of theory in this way is likely to contribute to interventions incorporating individual choice, agency, situated social and cultural influences and peer support as important dimensions, in order to reduce the harm associated with tobacco smoking. This is in contrast, but may be incorporated into, previous models of smoking relapse prevention, which were primarily psychological, focused on motivation and cue-driven behaviour [34].

A limitation of our work is that the data and thus conclusions are drawn from a cross-sectional qualitative sample. Our methods sought to answer open exploratory questions about the experience of e-cigarette use and patterns of use that may or may not support smoking relapse prevention. Although purposive, we matched our sample using a sampling frame to a nationally representative sample of UK quitters [35]. Despite our best efforts, the sample under-represents older populations of ex-smokers, those from lower socio-economic groups and ethnic minorities. Fully relapsed smokers we found ‘hard to reach’, perhaps due to shame and stigma associated with relapsed tobacco use. However, we are able to draw conclusions about relapse prevention in the context of vaping, since many of our sample reported experiencing relapse after initiating e-cigarette use, but had subsequently gone on to achieve abstinence through experimentation with different devices and vaping setups. Thus, although our findings are not statistically generalisable, we are reassured that they have transferability and could be reliably confirmed.

There was a divide in our sample between those who intended to continue to use e-cigarettes and those who eventually wanted to stop using nicotine altogether, as supported by other qualitative work [32]. This demonstrates some level of engagement with wider public health debates about the potential long-term utility of vaping as a harm reduction approach for smoking relapse prevention versus concerns about long-term use—the duality of e-cigarettes delivering nicotine as both a ‘poison’ and a ‘remedy’ [8]. Future research might usefully explore these stated intentions against long-term outcomes. A larger quantitative sample could explore associations over time between devices, patterns of use and abstinence outcomes, as we hypothesise based on our qualitative data that devices, e-liquids, patterns of use and stated intentions may all potentially impact on long-term tobacco abstinence. Eventually, we propose that future research might formally test an e-cigarette-based relapse prevention intervention, which critically must incorporate consumer choice and peer support beyond the intervention of the e-cigarette.

## Conclusions

This paper presents novel qualitative data on patterns of vaping over time and suggests potential factors, drawing on participant perspectives, in supporting long-term tobacco smoking abstinence. Findings show that, for this sample, e-cigarettes are a unique innovation supporting smoking relapse prevention. E-cigarettes are particularly attractive to ex-smokers, as having the right vaping setup is intrinsically satisfying and pleasurable. For many, vaping comes to be preferred over time to tobacco smoking, particularly as the user becomes more experienced and experiments until they are able to find an individualised setup that meets their needs. For some, e-cigarettes can substitute the physical, psychological, social, cultural and identity-related dimensions that were previously enjoyed about tobacco smoking, and thus may uniquely support long-term smoking relapse prevention. Many vapers were happy with their identity as a vaper and comfortable with nicotine addiction, but others saw e-cigarettes as a short-term smoking cessation intervention and eventually aimed to quit vaping as well as tobacco smoking. Vaping attracted some ex-smokers who had never intended to quit smoking and offered a viable and long-term alternative to tobacco smoking that was experienced as supporting relapse prevention.
